# Tris(β-ketoiminato)ruthenium(III) – Structural and electronic data of the neutral, oxidized and reduced forms

**DOI:** 10.1016/j.dib.2019.104833

**Published:** 2019-11-18

**Authors:** Jeanet Conradie

**Affiliations:** Department of Chemistry, PO Box 339, University of the Free State, Bloemfontein, 9300, South Africa

**Keywords:** Bidentate N,O ligands, (Z)-4-Aminopent-3-en-2-one, Ruthenium(III), DFT, Low-spin

## Abstract

In this data article, density functional theory (DFT) calculated data for the optimized geometries and electronic structure data of the neutral, oxidized and reduced forms of the *fac* and *mer* isomers of tris(amino-pent-3-en-2-onato-N,O)ruthenium(III) as representative example of tris(β-ketoiminato)ruthenium(III) complexes is provided. Energy-level diagrams of the neutral, oxidized and reduced molecules, show the effect on the molecular energy levels and the electron occupation of the frontier orbitals, when the neutral complex is oxidized or reduced. The DFT calculated data also confirms the spin state of the molecules and show that the *fac* and *mer* isomers of tris(amino-pent-3-en-2-onato-N,O)ruthenium(III) are equi-energetic.

**Table:** Specifications Table

Subject	Chemistry
Specific subject area	Computational chemistry.
Type of data	Table Image Figure
How data were acquired	Electronic structure calculations, using the Gaussian 2016 programme.
Data format	Raw Analyzed
Parameters for data collection	Suitable xyz coordinates for the input geometries were constructed using CHEMCRAFT. The input coordinates were used in the input file of the DFT program, example input files are provided in the supplementary information.
Description of data collection	Data were collected from DFT output files
Data source location	Department of Chemistry, University of the Free State, Nelson Mandela Street, Bloemfontein, South Africa
Data accessibility	With the article
Related research article	Tankiso Lawrence Ngake, Johannes. H. Potgieter and Jeanet Conradie, Tris(β-ketoiminato)ruthenium(III) complexes: Electrochemical and computational chemistry study. Electrochimica Acta 320 (2019) 134635 DOI 10.1016/j.electacta.2019.134635.

**Table:** 

**Value of the Data** • DFT calculated optimized structural data (coordinates) of tris(β-ketoiminato)ruthenium(III) complexes are provided for structural and computational chemistry researchers. • Data provide the geometrical and electronic structure of tris(β-ketoiminato)ruthenium(III) complexes. • This data can be used to determine the density functional theory calculated lowest energy spin states of the neutral, oxidized and reduced forms of tris(β-ketoiminato)ruthenium(III) complexes. • This data visualizes the molecular orbitals involved in oxidation and reduction of tris(β-ketoiminato)ruthenium(III) complexes. • This data provides density functional theory calculated molecular orbital energy level diagrams, for the neutral, oxidized and reduced forms of tris(β-ketoiminato)ruthenium(III) complexes. • This data provides density functional theory calculated ionization potential and electron affinity for *fac* and *mer* isomers of tris(amino-pent-3-en-2-onato-N,O)ruthenium(III). • This data can be used to understand the change in electron occupation and frontier molecular orbital energies, during reduction and oxidation of tris(β-ketoiminato)ruthenium(III) complexes.

## 1 Data

**Fig. 1:**
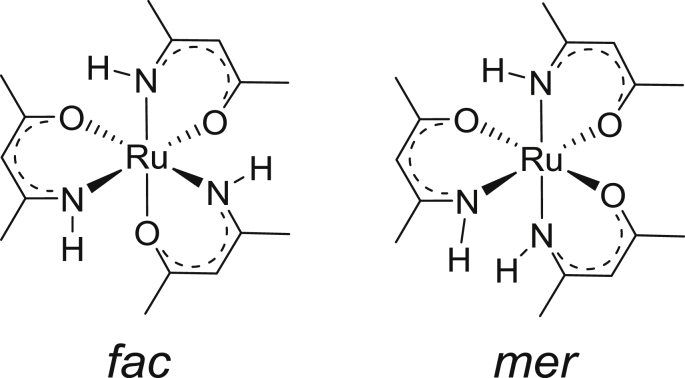
Structure of *fac* and *mer* tris(amino-pent-3-en-2-onato-N,O)ruthenium(III).

**Fig. 2:**
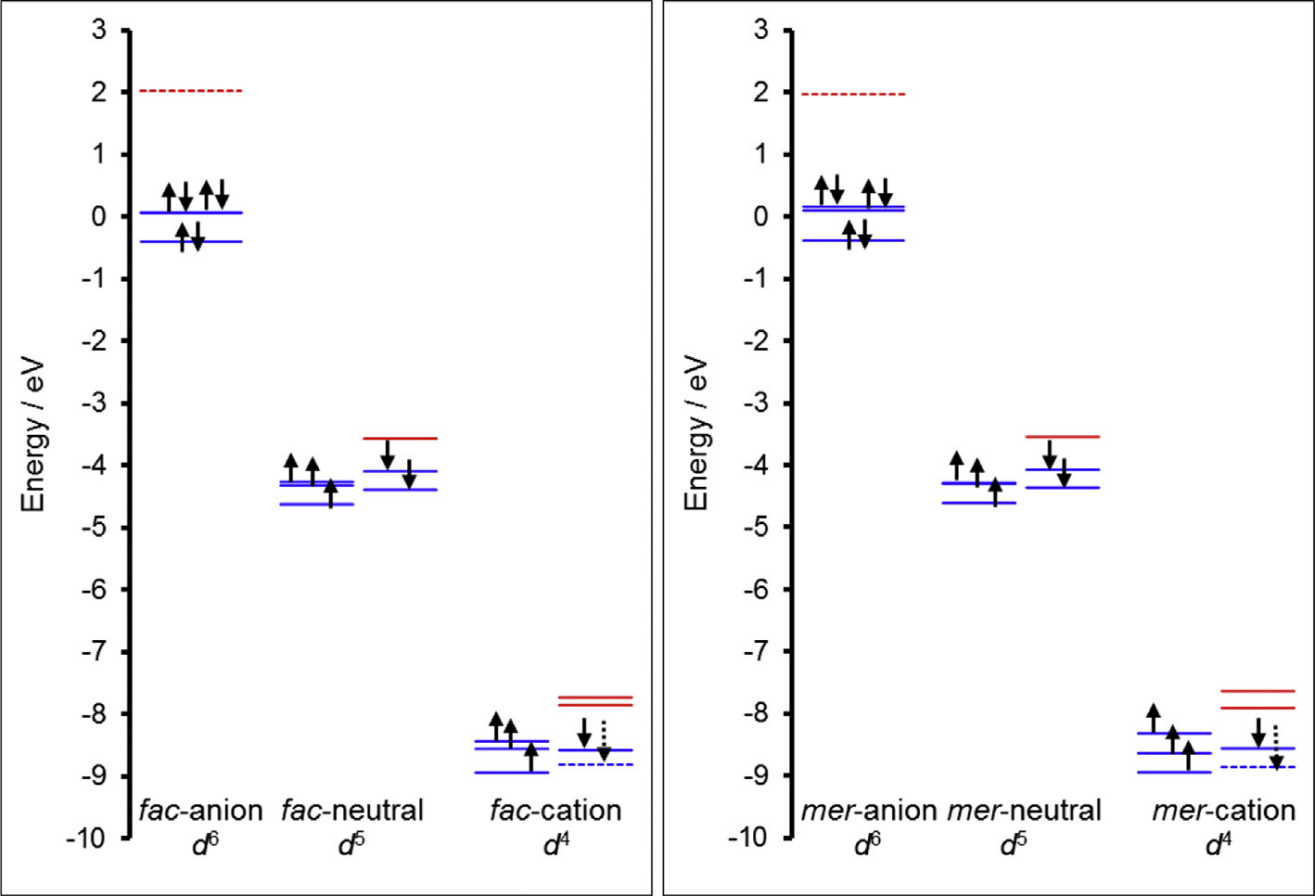
The OLYP/TZP Kohn-Sham MO energy level (in eV, on the y-axis) diagrams of the frontier MOs, for all three forms of *fac* (left) and *mer* (right) of tris(amino-pent-3-en-2-onato-N,O)ruthenium(III), namely the reduced (anion, left), neutral (middle) and oxidized (cation, right) forms. The energy levels of filled MOs are shown in solid blue (for Ru-d based MOs) or dotted blue (for ligand based MOs), and the energy levels of empty MOs in red (solid red Ru-d-based, dotted red ligand based). The arrows indicate the α-electrons (up spin) and β electrons (down spin), solid arrows indicate mainly Ru-d based MOs and the dotted arrow a mainly ligand based MO.

**Fig. 3:**
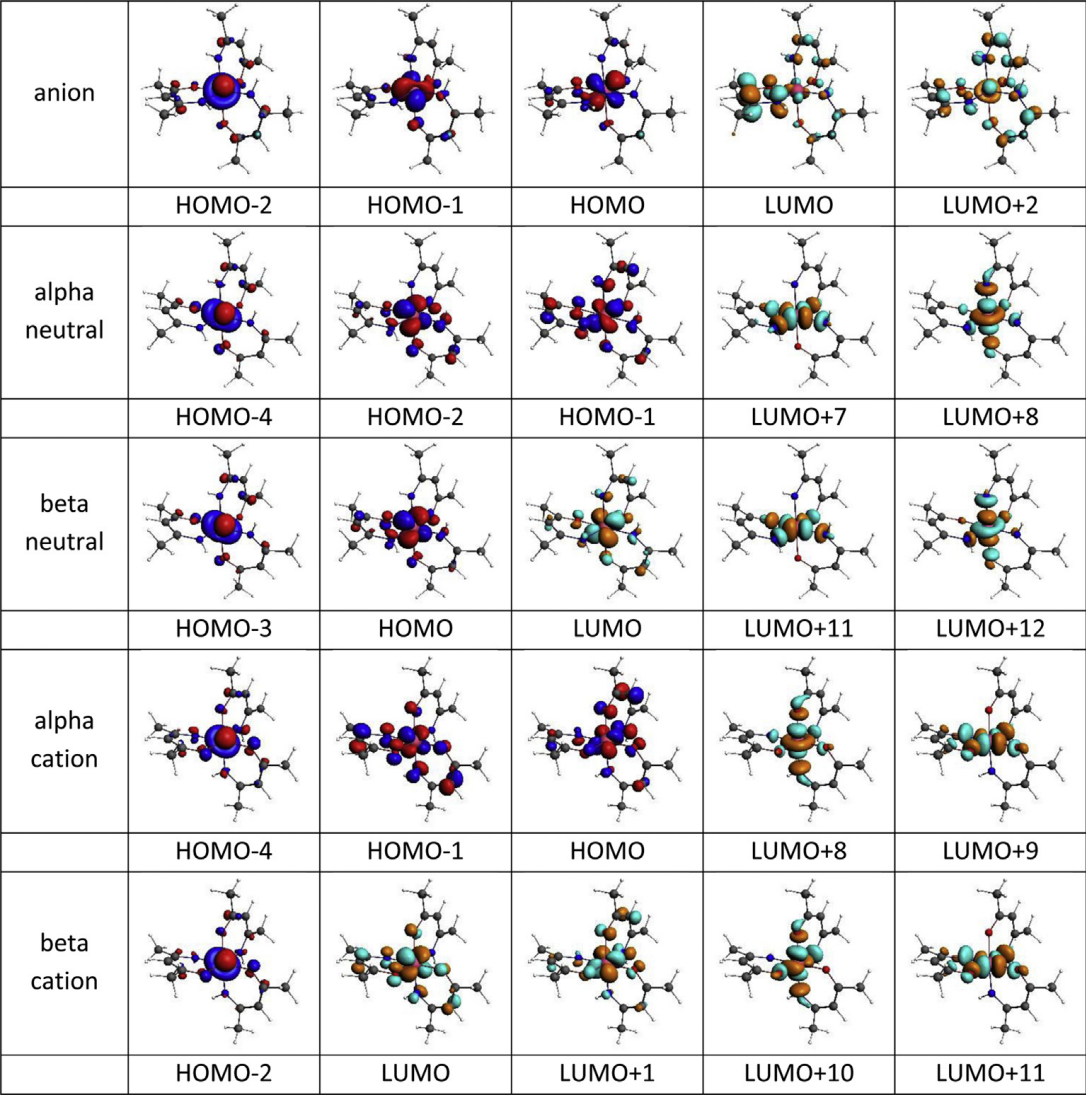
The BP86-D3/TZ2P(C,H,O,N)/ZORA/TZ2P(Ru) metal d-based anti-bonding MOs *fac* tris(amino-pent-3-en-2-onato-N,O)ruthenium(III. Contour = 0.06 e/Å^3^.

**Fig. 4:**
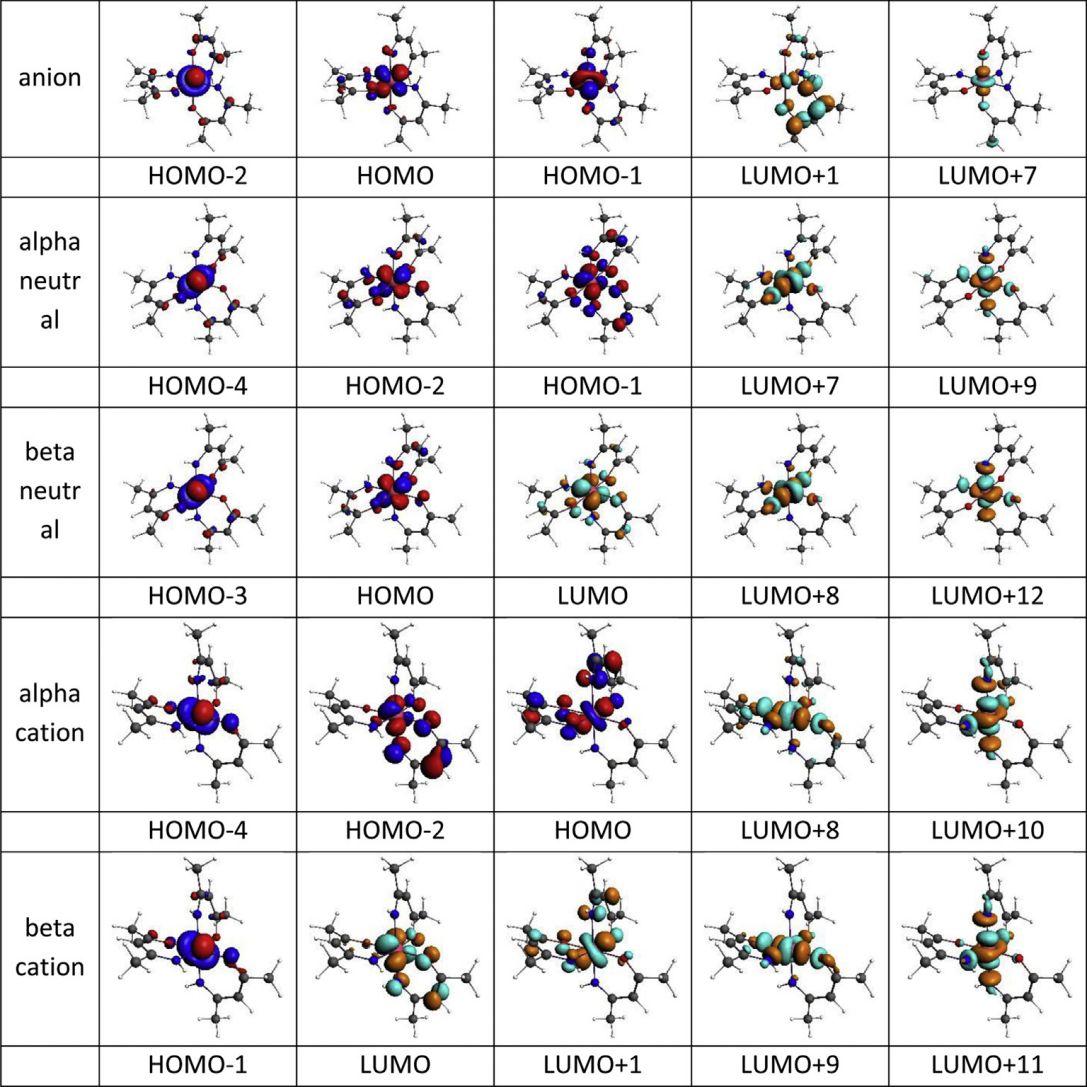
The BP86-D3/TZ2P(C,H,O,N)/ZORA/TZ2P(Ru) metal d-based anti-bonding MOs *mer* tris(amino-pent-3-en-2-onato-N,O)ruthenium(III. Contour = 0.06 e/Å^3^.

This data article provides the density functional theory (DFT) calculated geometrical and electronic structure data of the neutral, oxidized and reduced forms of the *fac* and *mer* isomers of tris(amino-pent-3-en-2-onato-N,O)ruthenium(III), **1** (Fig. 1), as representative example of tris(β-ketoiminato)ruthenium(III) complexes, that can electrochemically be oxidized and reduced [1]. Table 1 provides the DFT calculated energies for the different spin states possible for **1**, namely ½, 3/2 or 5/2, obtained by different DFT methods. The data shows that the neutral d^5^ molecule has a low spin state of ½, therefore contains one unpaired electron, as is experimentally observed for Ru(III) complexes [2,3]. The data in Table 1 further shows that the *fac* and *mer* isomers of **1** are near equi-energetic, thus both isomers are energetically possible. The data in Table 2 shows that the oxidized tris(β-ketoiminato)ruthenium(III) complexes are doublets (S = 1, two unpaired electrons), while the reduced complexes are singlets (S = 0, no unpaired electrons). The data in Table 3 provides the density functional theory calculated ionization potential and electron affinity for *fac* and *mer* isomers of tris(amino-pent-3-en-2-onato-N,O)ruthenium(III). Fig. 2 illustrates the effect on the energy of the molecular energy levels and the electron occupation of the frontier orbitals, when the neutral complex is oxidized or reduced for the *fac* and *mer* isomers of **1** respectively. The mainly d-based anti-bonding molecular orbitals of the *fac* and *mer* isomers of **1** are shown in Fig. 3 and Fig. 4 respectively.

**Table 1:** Relative energies (ΔE) for different spin states (S) of *fac* and *mer* tris(amino-pent-3-en-2-onato-N,O)ruthenium(III), as calculated with the indicated functional.

Isomer	S	B3LYP^a^	B3LYP-D3^a^	PBE-D2	OLYP-D3	BP86-D3	PW91-D3
*fac*	½	0.02	0.00	0.00	0.00	0.00	0.00
	3/2	1.93	2.38	1.97	1.90	1.94	1.97
	5/2	3.27	3.80	3.58	3.09	3.41	3.52
*mer*	½	0.00	0.06	0.03	0.03	0.01	0.01
	3/2	1.59	2.07	1.68	1.41	1.67	1.71
	5/2	3.09	3.65	3.45	2.94	3.33	3.39

a From Ref. [1].

**Table 2:** Relative energies (ΔE) for different spin states (S), of reduced and oxidized *fac* and *mer* tris(amino-pent-3-en-2-onato-N,O)ruthenium(III), calculated with the indicated functional.

	S	B3LYP	B3LYP-D3	PBE-D2	BP86-D3	OLYP-D3	PW91-D3
*Fac* anion	0	0.00	0.00	0.00	0.00	0.00	0.00
	1	1.56	1.57	1.55	1.53	1.57	1.54
*Mer* anion	0	0.02	0.07	0.07	0.05	0.08	0.05
	1	1.51	1.53	1.53	1.51	1.54	1.53
*Fac* cation	0	0.20	0.15	0.00	0.01	0.03	0.03
	1	0.01	0.02	0.01	0.00	0.00	0.00
*Mer* cation	0	0.26	0.22	0.10	0.07	0.12	0.05
	1	0.00	0.00	0.03	0.00	0.03	0.01

**Table 3:** Ionization potential (IP) and electron affinity (EA) of *fac* and *mer* tris(amino-pent-3-en-2-onato-N,O)ruthenium(III), calculated with the indicated functional.

	B3LYP	B3LYP-D3	PBE-D2	BP86-D3	OLYP-D3	PW91
IP *fac*	5.80	5.83	5.68	5.86	5.59	5.83
IP *mer*	5.82	5.82	5.66	5.86	5.60	5.83
EA *fac*	1.21	1.18	1.36	1.62	1.36	1.60
EA *mer*	1.17	1.15	1.33	1.57	1.32	1.55

## 2 Experimental design, materials, and methods

Density functional theory (DFT) calculations were performed in the gas phase on the neutral, oxidized and reduced compounds using the Gaussian 16 package [4] and the Amsterdam Density Functional (ADF) 2018 programme [5]. The data presented is obtained by different DFT methods (functional and basis set combination): (i) B3LYP functional [6,7] and the triple-ζ basis set 6-311G(d,p) on all atoms except for Ru where the Stuttgart/Dresden (SDD) pseudopotential was used to describe the metal electronic core, while the metal valence electrons were described using the def2-TZVPP basis set [8], (ii) B3LYP-D3/6-311G(d,p)/def2-TZVPP/SDD (B3LYP with the Grimme empirical dispersion correction D3) [9]), (iii) PBE-D2 (PBE with the Grimme empirical dispersion correction D3) with 6-311G(d,p) on all nonmetallic atoms and LANL2DZ with included ECP [(c), (d), [10], (a), (b)] augmented with one f-polarization function (1.235) [11] on Ru, (iv) OLYP (Handy-Cohen and Lee-Yang-Parr) [6,[12], [13], [14]] with the TZ2P (Triple ζ double polarized) basis set on all atoms (C,H,O,N) except for Ru where the ZORA/TZ2P was used, (v) BP86/TZ2P(C,H,O,N)/ZORA/TZ2P(Ru) [15,16], (vi) PW91/TZ2P(C,H,O,N)/ZORA/TZ2P(Ru) (Perdew-Wang 1991 [[b], [17], [a]]. Input coordinates were constructed using ChemCraft [18]. ADF Graphical User Interface (GUI) and Chemcraft were used to visualize the output files. The optimized coordinates, as well as example input files, are provided in the supplementary information.

## Appendix A Supplementary data

Multimedia component 1 Input and output files and optimized coordinates of DFT calculations. Multimedia component 1
