# Supplementary material for: Tris(β-ketoiminato)ruthenium(III) – Structural and electronic data of the neutral, oxidized and reduced forms
Source: Data Brief. 2019 Nov 18;28:104833. doi: 10.1016/j.dib.2019.104833 (PMC6889778; doi:10.1016/j.dib.2019.104833)
Supplement: Multimedia component 1 — Input and output files and optimized coordinates of DFT calculations. [file mmc1.docx]

**Data Article**

**Title**: Tris(β-ketoiminato)ruthenium(III) – structural and electronic data of the neutral, oxidized and reduced forms.

**Author**: Jeanet Conradie

**Affiliation**: Department of Chemistry, PO Box 339, University of the Free State, Bloemfontein, 9300, South Africa

**Contact email**: conradj@ufs.ac.za

Supporting information

Table of Contents

[**Data Article** 1](#_Toc22482586)

[Example input files 2](#_Toc22482587)

[i) OLYP-D3 with ADF 2](#_Toc22482588)

[ii) BP86-D3 with ADF 3](#_Toc22482589)

[iii) PW91-D3 with ADF 5](#_Toc22482590)

[iv) B3LYP with Gaussian 7](#_Toc22482591)

[v) B3LYP-D3 with Gaussian 8](#_Toc22482592)

[vi) PBE-D2 with Gaussian 9](#_Toc22482593)

[Optimized Cartesian coordinates (Å) 12](#_Toc22482594)

[1. *fac* tris(amino-pent-3-en-2-onato-N,O)ruthenium(III) S = ½ BP86/TZ2P 12](#_Toc22482595)

[2. *mer* tris(amino-pent-3-en-2-onato-N,O)ruthenium(III) S = ½ BP86/TZ2P 12](#_Toc22482596)

# Example input files

## OLYP-D3 with ADF

Title *mer* tris(amino-pent-3-en-2-onato-N,O)ruthenium(III)

COMMENT

ruthenium(III) is low spin S = 1/2

END

UNITS

length angstrom

END

Atoms Cartesian

Ru 0.035286000 0.057071000 -0.026963000

O 0.083945000 -1.535863000 1.108923000

N -1.331413000 -0.715650000 -1.137055000

H -1.571410000 -0.217624000 -1.989545000

O -1.273750000 0.816264000 1.184618000

N 0.003072000 1.640181000 -1.119665000

H 0.759398000 1.733403000 -1.792936000

N 1.460067000 0.777329000 1.140434000

H 1.142308000 1.331999000 1.930133000

O 1.403936000 -0.669861000 -1.235815000

C 2.749640000 0.577591000 1.094427000

C 3.365120000 -0.171034000 0.049465000

C 2.680503000 -0.730335000 -1.020901000

C -0.730739000 -2.527686000 1.065283000

C -1.727325000 -2.727304000 0.101384000

C -1.966218000 -1.861304000 -0.989063000

C -2.024556000 1.842494000 0.943937000

C -1.886548000 2.719694000 -0.129215000

C -0.838909000 2.647435000 -1.087771000

C -2.957530000 -2.290704000 -2.052768000

H -3.759542000 -2.900262000 -1.626324000

H -3.408021000 -1.427218000 -2.552911000

H -2.449943000 -2.896153000 -2.815287000

C -0.531541000 -3.550696000 2.167665000

H -0.681038000 -3.070411000 3.142093000

H -1.215356000 -4.398578000 2.079985000

H 0.501144000 -3.918160000 2.145141000

C 3.440477000 -1.496259000 -2.087210000

H 3.137505000 -2.550458000 -2.064969000

H 3.183266000 -1.109903000 -3.080135000

H 4.522414000 -1.435975000 -1.946424000

C 3.648640000 1.148411000 2.171769000

H 3.087684000 1.744983000 2.896967000

H 4.154581000 0.337488000 2.709795000

H 4.428325000 1.779127000 1.729219000

C -0.666653000 3.803753000 -2.051189000

H -0.033096000 3.533576000 -2.901468000

H -1.636001000 4.139582000 -2.433884000

H -0.205991000 4.658164000 -1.538568000

C -3.106178000 2.070980000 1.980836000

H -3.701740000 2.962226000 1.768731000

H -3.767741000 1.197364000 2.019247000

H -2.652433000 2.172229000 2.973860000

H -2.324009000 -3.630425000 0.168479000

H -2.568516000 3.560858000 -0.189393000

H 4.441563000 -0.296998000 0.087102000

end

SYMMETRY nosym

CHARGE 0 1

unrestricted

GEOMETRY

Iterations 500

End

SCF

Iterations 500

END

BASIS

Type TZ2P

Core none

END

XC

gga OLYP

DISPERSION Grimme3

END

endinput

## BP86-D3 with ADF

Title *mer* tris(amino-pent-3-en-2-onato-N,O)ruthenium(III)

COMMENT

ruthenium(III) is low spin S = 1/2

END

UNITS

length angstrom

END

Atoms Cartesian

Ru 0.035286000 0.057071000 -0.026963000

O 0.083945000 -1.535863000 1.108923000

N -1.331413000 -0.715650000 -1.137055000

H -1.571410000 -0.217624000 -1.989545000

O -1.273750000 0.816264000 1.184618000

N 0.003072000 1.640181000 -1.119665000

H 0.759398000 1.733403000 -1.792936000

N 1.460067000 0.777329000 1.140434000

H 1.142308000 1.331999000 1.930133000

O 1.403936000 -0.669861000 -1.235815000

C 2.749640000 0.577591000 1.094427000

C 3.365120000 -0.171034000 0.049465000

C 2.680503000 -0.730335000 -1.020901000

C -0.730739000 -2.527686000 1.065283000

C -1.727325000 -2.727304000 0.101384000

C -1.966218000 -1.861304000 -0.989063000

C -2.024556000 1.842494000 0.943937000

C -1.886548000 2.719694000 -0.129215000

C -0.838909000 2.647435000 -1.087771000

C -2.957530000 -2.290704000 -2.052768000

H -3.759542000 -2.900262000 -1.626324000

H -3.408021000 -1.427218000 -2.552911000

H -2.449943000 -2.896153000 -2.815287000

C -0.531541000 -3.550696000 2.167665000

H -0.681038000 -3.070411000 3.142093000

H -1.215356000 -4.398578000 2.079985000

H 0.501144000 -3.918160000 2.145141000

C 3.440477000 -1.496259000 -2.087210000

H 3.137505000 -2.550458000 -2.064969000

H 3.183266000 -1.109903000 -3.080135000

H 4.522414000 -1.435975000 -1.946424000

C 3.648640000 1.148411000 2.171769000

H 3.087684000 1.744983000 2.896967000

H 4.154581000 0.337488000 2.709795000

H 4.428325000 1.779127000 1.729219000

C -0.666653000 3.803753000 -2.051189000

H -0.033096000 3.533576000 -2.901468000

H -1.636001000 4.139582000 -2.433884000

H -0.205991000 4.658164000 -1.538568000

C -3.106178000 2.070980000 1.980836000

H -3.701740000 2.962226000 1.768731000

H -3.767741000 1.197364000 2.019247000

H -2.652433000 2.172229000 2.973860000

H -2.324009000 -3.630425000 0.168479000

H -2.568516000 3.560858000 -0.189393000

H 4.441563000 -0.296998000 0.087102000

end

SYMMETRY nosym

CHARGE 0 1

unrestricted

GEOMETRY

Iterations 500

End

SCF

Iterations 500

END

BASIS

Type TZ2P

Core none

END

XC

gga OLYP

DISPERSION Grimme3

END

endinput

## PW91-D3 with ADF

Title *mer* tris(amino-pent-3-en-2-onato-N,O)ruthenium(III)

COMMENT

ruthenium(III) is low spin S = 1/2

END

UNITS

length angstrom

END

Atoms Cartesian

Ru 0.035286000 0.057071000 -0.026963000

O 0.083945000 -1.535863000 1.108923000

N -1.331413000 -0.715650000 -1.137055000

H -1.571410000 -0.217624000 -1.989545000

O -1.273750000 0.816264000 1.184618000

N 0.003072000 1.640181000 -1.119665000

H 0.759398000 1.733403000 -1.792936000

N 1.460067000 0.777329000 1.140434000

H 1.142308000 1.331999000 1.930133000

O 1.403936000 -0.669861000 -1.235815000

C 2.749640000 0.577591000 1.094427000

C 3.365120000 -0.171034000 0.049465000

C 2.680503000 -0.730335000 -1.020901000

C -0.730739000 -2.527686000 1.065283000

C -1.727325000 -2.727304000 0.101384000

C -1.966218000 -1.861304000 -0.989063000

C -2.024556000 1.842494000 0.943937000

C -1.886548000 2.719694000 -0.129215000

C -0.838909000 2.647435000 -1.087771000

C -2.957530000 -2.290704000 -2.052768000

H -3.759542000 -2.900262000 -1.626324000

H -3.408021000 -1.427218000 -2.552911000

H -2.449943000 -2.896153000 -2.815287000

C -0.531541000 -3.550696000 2.167665000

H -0.681038000 -3.070411000 3.142093000

H -1.215356000 -4.398578000 2.079985000

H 0.501144000 -3.918160000 2.145141000

C 3.440477000 -1.496259000 -2.087210000

H 3.137505000 -2.550458000 -2.064969000

H 3.183266000 -1.109903000 -3.080135000

H 4.522414000 -1.435975000 -1.946424000

C 3.648640000 1.148411000 2.171769000

H 3.087684000 1.744983000 2.896967000

H 4.154581000 0.337488000 2.709795000

H 4.428325000 1.779127000 1.729219000

C -0.666653000 3.803753000 -2.051189000

H -0.033096000 3.533576000 -2.901468000

H -1.636001000 4.139582000 -2.433884000

H -0.205991000 4.658164000 -1.538568000

C -3.106178000 2.070980000 1.980836000

H -3.701740000 2.962226000 1.768731000

H -3.767741000 1.197364000 2.019247000

H -2.652433000 2.172229000 2.973860000

H -2.324009000 -3.630425000 0.168479000

H -2.568516000 3.560858000 -0.189393000

H 4.441563000 -0.296998000 0.087102000

end

SYMMETRY nosym

CHARGE 0 1

unrestricted

GEOMETRY

Iterations 500

End

SCF

Iterations 500

END

BASIS

Type TZ2P

Core none

END

XC

gga PW91

DISPERSION Grimme3

END

endinput

## B3LYP with Gaussian

%chk=RuL2_3_mer_S12_b3lyp.chk

#p b3lyp/genecp

opt

Comment: *mer* tris(amino-pent-3-en-2-onato-N,O)ruthenium(III)

0 2

44 0.063779000 0.138222000 -0.016870000

8 0.119823000 -1.549824000 1.135962000

7 -1.401273000 -0.678944000 -1.145231000

1 -1.745937000 -0.156921000 -1.944884000

8 -1.280757000 0.981441000 1.233289000

7 -0.022344000 1.807555000 -1.170113000

1 0.710536000 1.936168000 -1.861506000

7 1.578158000 0.870326000 1.186265000

1 1.314441000 1.489951000 1.945852000

8 1.486923000 -0.594513000 -1.256011000

6 2.834339000 0.531228000 1.169665000

6 3.397158000 -0.303028000 0.155422000

6 2.733810000 -0.784745000 -0.955710000

6 -0.642331000 -2.570993000 1.013442000

6 -1.631410000 -2.755670000 0.046476000

6 -1.969937000 -1.855074000 -0.988304000

6 -2.073930000 1.956782000 0.932843000

6 -1.975451000 2.781075000 -0.176685000

6 -0.929950000 2.747293000 -1.145409000

6 -3.021975000 -2.292990000 -1.986670000

1 -3.801287000 -2.885082000 -1.503516000

1 -3.491019000 -1.433454000 -2.471065000

1 -2.567237000 -2.915175000 -2.764287000

6 -0.397870000 -3.654040000 2.041956000

1 -0.548817000 -3.243132000 3.044064000

1 -1.053104000 -4.515310000 1.906685000

1 0.643433000 -3.982429000 1.981762000

6 3.480115000 -1.603441000 -1.983659000

1 3.011667000 -2.588024000 -2.070892000

1 3.401898000 -1.123295000 -2.963297000

1 4.533140000 -1.729282000 -1.729914000

6 3.768957000 1.032080000 2.248732000

1 3.258173000 1.700155000 2.944826000

1 4.171992000 0.187566000 2.815404000

1 4.617133000 1.564882000 1.810128000

6 -0.865705000 3.879891000 -2.146710000

1 -0.120754000 3.689518000 -2.921822000

1 -1.837035000 4.021341000 -2.628045000

1 -0.612453000 4.818738000 -1.645273000

6 -3.169721000 2.184525000 1.947853000

1 -3.810424000 3.027299000 1.686477000

1 -3.779664000 1.280600000 2.031923000

1 -2.725355000 2.362666000 2.931061000

1 -2.182356000 -3.686436000 0.079725000

1 -2.703803000 3.575993000 -0.271808000

1 4.449098000 -0.540154000 0.247902000

C N H O 0

6-311G(d,p)

****

Ru 0

def2tzvpp

****

Ru 0

SDD

## B3LYP-D3 with Gaussian

%chk=RuL2_3_mer_S12_b3lyp_D3.chk

#p b3lyp/genecp

EmpiricalDispersion=GD3

opt

Comment: *mer* tris(amino-pent-3-en-2-onato-N,O)ruthenium(III)

0 2

44 0.063779000 0.138222000 -0.016870000

8 0.119823000 -1.549824000 1.135962000

7 -1.401273000 -0.678944000 -1.145231000

1 -1.745937000 -0.156921000 -1.944884000

8 -1.280757000 0.981441000 1.233289000

7 -0.022344000 1.807555000 -1.170113000

1 0.710536000 1.936168000 -1.861506000

7 1.578158000 0.870326000 1.186265000

1 1.314441000 1.489951000 1.945852000

8 1.486923000 -0.594513000 -1.256011000

6 2.834339000 0.531228000 1.169665000

6 3.397158000 -0.303028000 0.155422000

6 2.733810000 -0.784745000 -0.955710000

6 -0.642331000 -2.570993000 1.013442000

6 -1.631410000 -2.755670000 0.046476000

6 -1.969937000 -1.855074000 -0.988304000

6 -2.073930000 1.956782000 0.932843000

6 -1.975451000 2.781075000 -0.176685000

6 -0.929950000 2.747293000 -1.145409000

6 -3.021975000 -2.292990000 -1.986670000

1 -3.801287000 -2.885082000 -1.503516000

1 -3.491019000 -1.433454000 -2.471065000

1 -2.567237000 -2.915175000 -2.764287000

6 -0.397870000 -3.654040000 2.041956000

1 -0.548817000 -3.243132000 3.044064000

1 -1.053104000 -4.515310000 1.906685000

1 0.643433000 -3.982429000 1.981762000

6 3.480115000 -1.603441000 -1.983659000

1 3.011667000 -2.588024000 -2.070892000

1 3.401898000 -1.123295000 -2.963297000

1 4.533140000 -1.729282000 -1.729914000

6 3.768957000 1.032080000 2.248732000

1 3.258173000 1.700155000 2.944826000

1 4.171992000 0.187566000 2.815404000

1 4.617133000 1.564882000 1.810128000

6 -0.865705000 3.879891000 -2.146710000

1 -0.120754000 3.689518000 -2.921822000

1 -1.837035000 4.021341000 -2.628045000

1 -0.612453000 4.818738000 -1.645273000

6 -3.169721000 2.184525000 1.947853000

1 -3.810424000 3.027299000 1.686477000

1 -3.779664000 1.280600000 2.031923000

1 -2.725355000 2.362666000 2.931061000

1 -2.182356000 -3.686436000 0.079725000

1 -2.703803000 3.575993000 -0.271808000

1 4.449098000 -0.540154000 0.247902000

C N H O 0

6-311G(d,p)

****

Ru 0

def2tzvpp

****

Ru 0

SDD

## PBE-D2 with Gaussian

%chk=RuL2_3_mer_S12_PBEPBE_gas.chk

#p PBEPBE/genecp

opt=(tight)

scf=(conver=8)

empiricaldispersion=GD2

extrabasis pseudo=read

Comment: *mer* tris(amino-pent-3-en-2-onato-N,O)ruthenium(III)

0 2

44 0.115412000 0.181661000 -0.006019000

8 0.189008000 -1.521709000 1.141458000

7 -1.367377000 -0.587517000 -1.119999000

1 -1.726820000 -0.031099000 -1.902574000

8 -1.257949000 0.979594000 1.262609000

7 0.018433000 1.836056000 -1.146373000

1 0.775074000 2.006095000 -1.816572000

7 1.627781000 0.893499000 1.193055000

1 1.380480000 1.546880000 1.941506000

8 1.519196000 -0.580497000 -1.271183000

6 2.882318000 0.509237000 1.192470000

6 3.422248000 -0.354311000 0.188426000

6 2.761222000 -0.811726000 -0.944877000

6 -0.602755000 -2.531758000 1.013288000

6 -1.622875000 -2.676067000 0.059217000

6 -1.972829000 -1.755751000 -0.959131000

6 -2.110354000 1.897395000 0.908136000

6 -2.038652000 2.691114000 -0.236220000

6 -0.961423000 2.718426000 -1.173448000

6 -3.068151000 -2.149283000 -1.927802000

1 -3.856911000 -2.722668000 -1.417862000

1 -3.523630000 -1.260819000 -2.392894000

1 -2.657784000 -2.785483000 -2.730641000

6 -0.360029000 -3.642685000 2.012957000

1 -0.481223000 -3.248947000 3.035333000

1 -1.044734000 -4.490501000 1.867200000

1 0.680365000 -3.995152000 1.922136000

6 3.501294000 -1.648907000 -1.963521000

1 3.002507000 -2.627189000 -2.062836000

1 3.453532000 -1.160319000 -2.950589000

1 4.553388000 -1.803645000 -1.684872000

6 3.826793000 0.996750000 2.268640000

1 3.316860000 1.661935000 2.982038000

1 4.237971000 0.137865000 2.824448000

1 4.678345000 1.537364000 1.823745000

6 -0.932355000 3.841665000 -2.185667000

1 -0.174354000 3.662728000 -2.963754000

1 -1.916075000 3.947227000 -2.670795000

1 -0.707553000 4.801775000 -1.691209000

6 -3.241031000 2.090016000 1.892207000

1 -3.929862000 2.887687000 1.579595000

1 -3.798434000 1.144793000 1.998459000

1 -2.828258000 2.333777000 2.884764000

1 -2.198275000 -3.602827000 0.095200000

1 -2.823095000 3.437990000 -0.370570000

1 4.473234000 -0.629328000 0.292557000

C H O N 0

6-311G(d,p)

****

Ru 0

Lanl2dz

****

Ru 0

F 1 1.0

1.235 1.0

****

Ru 0

Lanl2dz

Eps=10

# Optimized Cartesian coordinates (Å)

# *fac* tris(amino-pent-3-en-2-onato-N,O)ruthenium(III) S = ½ BP86/TZ2P

# *mer* tris(amino-pent-3-en-2-onato-N,O)ruthenium(III) S = ½ BP86/TZ2P
